# Supplementary material for: A Multitechnique Study of C2H4 Adsorption on Fe3O4(001)
Source: J Phys Chem C Nanomater Interfaces. 2023 Sep 11;127(37):18378–88. doi: 10.1021/acs.jpcc.3c03684 (PMC10518864; doi:10.1021/acs.jpcc.3c03684)
Supplement: Supplementary file 1 — jp3c03684_si_001.pdf [file jp3c03684_si_001.pdf]

Supporting Information for
A Multi-Technique Study of C_2H_4 Adsorption on $Fe_3O_4(001)$

Lena Puntscher[1*], Panukorn Sombut[1*], Chunlei Wang[1], Manuel Ulreich[1], Jiri Pavelec[1], Ali Rafsanjani-Abbasi[1], Matthias Meier[1,2], Adam Lagin[1], Martin Setvin[1,3], Ulrike Diebold[1], Cesare Franchini[2,4], Michael Schmid[1] and Gareth S. Parkinson[1]

[1]Institute of Applied Physics, TU Wien, Vienna, Austria
[2]Faculty of Physics, Center for Computational Materials Science, University of Vienna, Vienna, Austria
[3]Department of Surface and Plasma Science, Faculty of Mathematics and Physics, Charles University, Prague, Czech Republic
[4]Dipartimento di Fisica e Astronomia, Università di Bologna, Bologna, Italy

Initial coverages for the TPD series shown in Fig. 1:
Figure 1 shows a series of TPD spectra for various initial coverages of (0.3, 0.6, 0.9, 1.2, 1.6, 2.1, 2.4, 2.7, 2.9, 3.0, 3.4, 3.9, 4.2, 4.6, 4.9, 5.8, 6.0, 6.7, 7.5, 8.4, 9.1, 12.1) C_2H_4 molecules per (√2×√2)R45° unit cell.

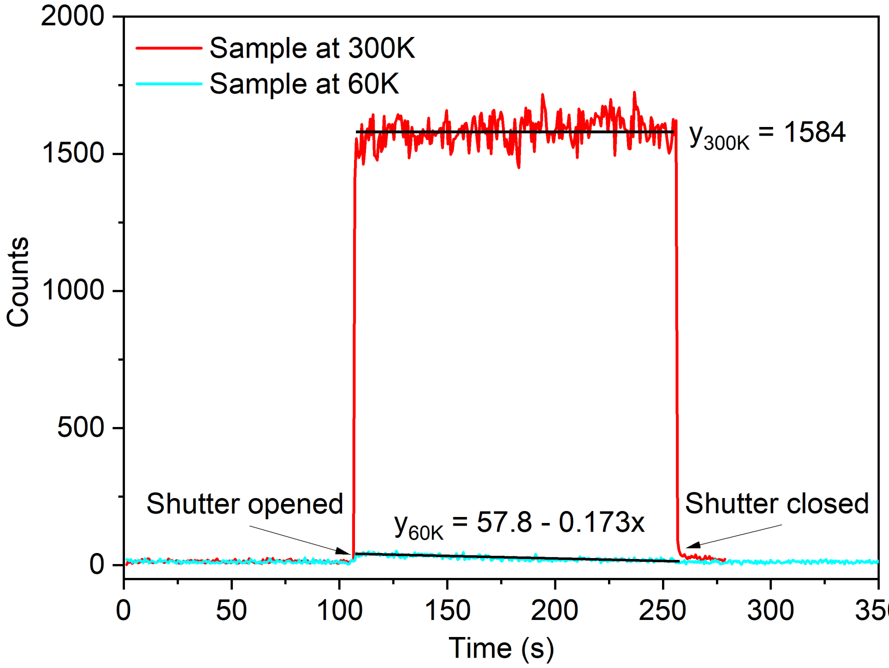

Figure S1: The sticking coefficient measurement performed here is based on the King and Wells approach[1]. For the reference of zero sticking, the as-prepared sample is held in UHV at 300 K, which is well above the temperature of any peaks observed in TPD (see Figure 1 in the main text). After a time of 105 s, the molecular beam shutter is opened and C_2H_4 molecules impinge on the sample at normal incidence. Molecules reflect from the surface and scatter into the vacuum system, and some are measured by the mass spectrometer. Ideally, the mass spectrometer is

positioned in a non line of sight geometry to prevent direct scattering into the mass spectrometer. In our setup, however, this is not possible as the angle between the mass spectrometer and molecular beam source is fixed at 30°. When this experiment is repeated at 60 K, the signal is much lower because most of the molecules are adsorbed at the sample surface. To determine the sticking coefficient, one has to calculate the difference between the two curves as a function of time and divide it by the background-corrected count rate at zero sticking, y_{300K}. In our measurement shown in Fig. S1, the signal acquired for the 300 K measurement is constant, as expected. The 60 K signal begins at 2.5% of the intensity (after subtraction of the background) and decreases approximately linearly to 0 (i.e. 100% sticking) after 150 seconds. The slow increase to 100% sticking is typical for such molecules on surfaces, and occurs because momentum transfer is maximised once molecules arrive at a surface already covered in similar molecules. The formulae shown in the figure result from a linear fit to the data for the duration of time that the shutter was open. A more thorough description of sticking coefficient measurements is contained within the work of Chen et al.[2].

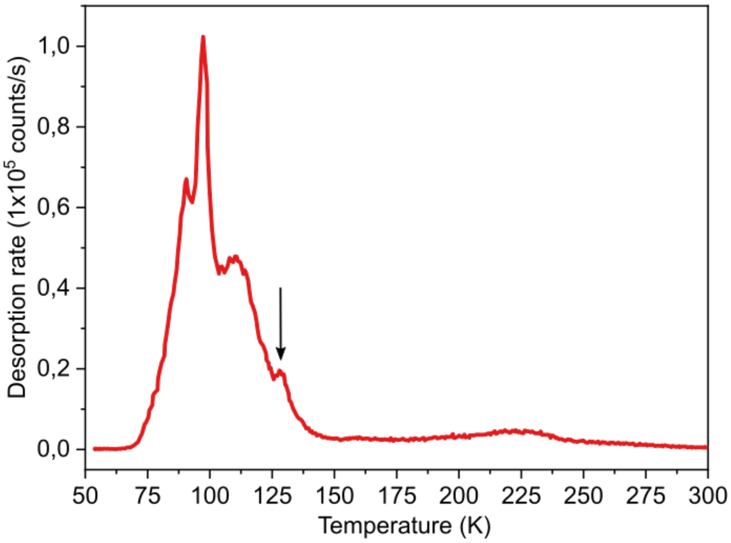

Figure S2: TPD curve for 4.1 C_2H_4/unit cell acquired on a sample previously utilized daily for surface science experiments in our setup for approximately 1 year. The data is similar to that obtained on a freshly installed sample (see Figure 1 in the main text), apart from an enhanced intensity of the shoulder at 125 K, which becomes a peak (see black arrow). This peak was previously observed with a similar intensity by Lee et al. [3]. One possible explanation for this peak is adsorption at α-Fe_2O_3 inclusions. De la Figuera and coworkers have shown that a typical annealing cycle with a partial pressure of 10^{-6} mbar O_2 leads to the growth of many hundreds of layers of virgin Fe_3O_4(001) surface [4, 5]. This is one of the reasons why this surface is relatively straightforward to prepare, but the iron required for this growth is obtained by oxidizing the sample overall. Rather than a homogeneous distribution of iron vacancies, the oxidation manifests in the growth of α-Fe_2O_3 inclusions, which grow along the <110> directions at the Fe_3O_4(001) surface. In extreme cases, these can be visible to the eye as a chequerboard appearance on the sample surface, as can be seen in Ref. 5. In the early stages it appears more as a matte appearance of the sample surface, compared the extremely polished appearance of the as-purchased samples.

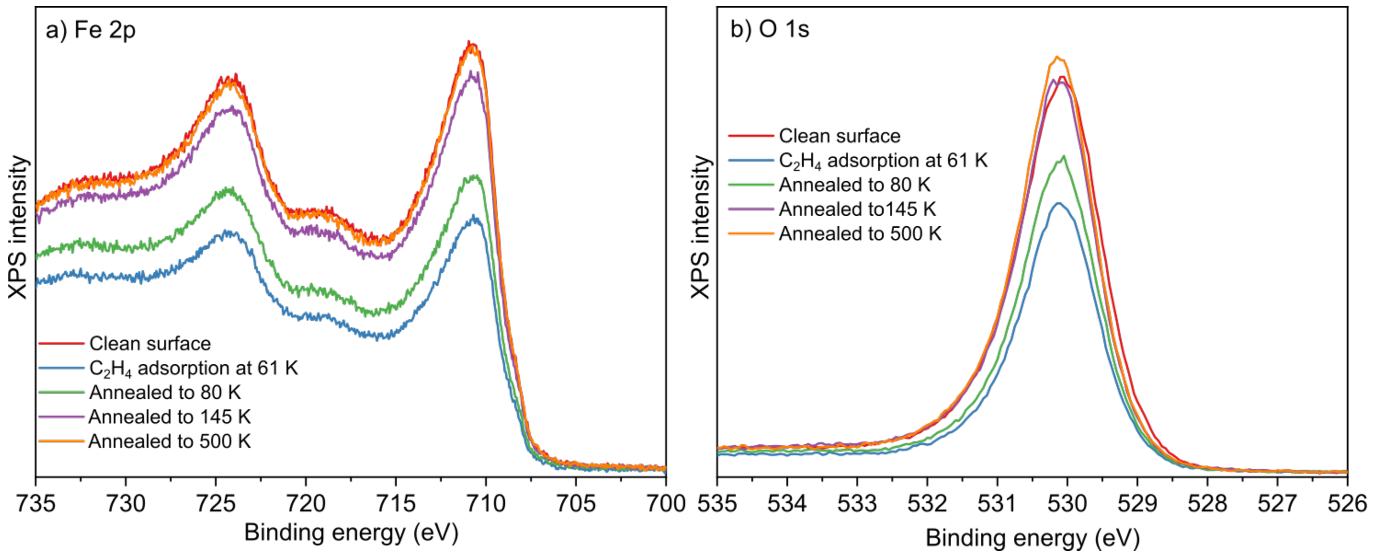

Figure S3: X-ray photoelectron spectroscopy data for the $C_2H_4/Fe_3O_4(001)$ system measured at 61 K after C_2H_4 adsorption and after several heating steps up to 500 K. In a) the Fe2p region is shown, in b) the O1s region.

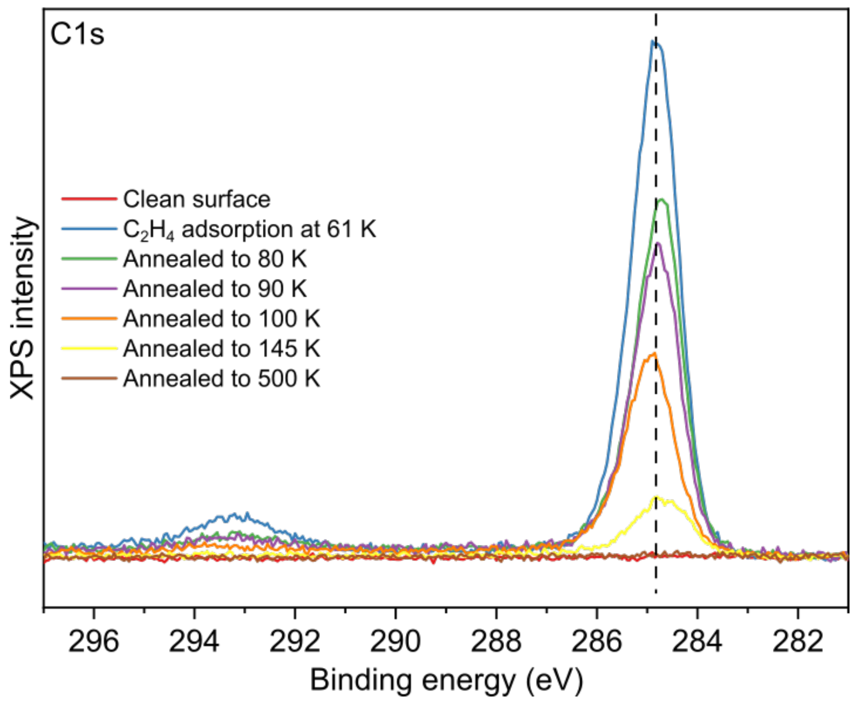

Figure S4: Grazing emission X-ray photoelectron spectroscopy data for the $C_2H_4/Fe_3O_4(001)$ system measured at 61 K after C_2H_4 adsorption and after several heating steps up to 500 K. A second peak shifted by 8.3 eV to higher binding energy from the C1s peak at 284.8 eV is due to a pi-3p Rydberg shake-up process.

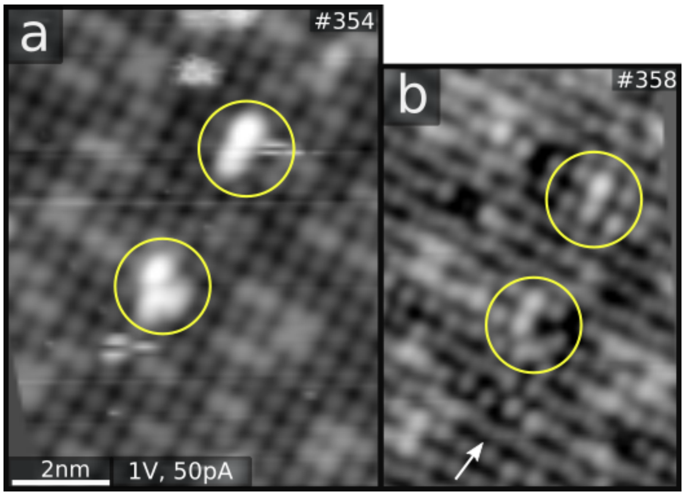

Figure S5: STM images acquired at $T = 74$ K of the $Fe_3O_4(001)$ surface during exposure to ethylene. In (a) ethylene molecules only adsorb at defect sites, while in (b) also the Fe^{3+} rows start to get occupied. There are still Fe^{3+} rows that appear unoccupied (marked with an arrow). The yellow circles show a defect site which is occupied by ethylene in both images. It is clearly visible in (b) that the ethylene molecules adsorbed at defect sites appear brighter than the ethylene molecules adsorbed at regular lattice sites.

References

(1) King, D. A.; Wells, M. G. Molecular beam investigation of adsorption kinetics on bulk metal targets: Nitrogen on tungsten. *Surface Science* **1972**, *29* (2), 454-482.
(2) Chen, L.; Smith, R. S.; Kay, B. D.; Dohnálek, Z. Adsorption of small hydrocarbons on rutile $TiO_2(110)$. *Surface Science* **2016**, *650*, 83-92.
(3) Lee, C. J.; Sharp, M. A.; Smith, R. S.; Kay, B. D.; Dohnálek, Z. Adsorption of ethane, ethene, and ethyne on reconstructed $Fe_3O_4(001)$. *Surface Science* **2021**, *714*, 121932.
(4) Nie, S.; Starodub, E.; Monti, M.; Siegel, D. A.; Vergara, L.; El Gabaly, F.; Bartelt, N. C.; de la Figuera, J.; McCarty, K. F. Insight into magnetite's redox catalysis from observing surface morphology during oxidation. *Journal of the American Chemical Society* **2013**, *135* (27), 10091-10098.
(5) McCarty, K. F.; Monti, M.; Nie, S.; Siegel, D. A.; Starodub, E.; El Gabaly, F.; McDaniel, A. H.; Shavorskiy, A.; Tyliszczak, T.; Bluhm, H.; Bartelt, N. C.; de la Figuera, J. Oxidation of Magnetite(100) to Hematite Observed by in Situ Spectroscopy and Microscopy. *The Journal of Physical Chemistry C* **2014**, *118* (34), 19768-19777.
